# Understanding the Well-Being of Older Chinese Immigrants in Relation to Green Spaces: A Gold Coast Study (Australia)

**DOI:** 10.3389/fpsyg.2020.551213

**Published:** 2020-10-28

**Authors:** Siyao Gao, Caryl Bosman, Karine Dupre

**Affiliations:** School of Engineering and Built Environment, Griffith University, Gold Coast, QLD, Australia

**Keywords:** green space, values, accessibility, older Chinese immigrants, well-being

## Abstract

In recognition of the aging population and the importance of health-supporting urban environments, including urban green spaces, to maintain well-being, scholars and policymakers have increasingly investigated the associations between urban green spaces and the well-being of older people. However, few studies specifically investigate minority older groups such as those with diverse cultural backgrounds, and many studies often ignore the design attributes of green spaces which may contribute to the well-being of those in such groups. In order to address these gaps, this paper explores how green spaces influence the well-being of older Chinese immigrants. This case study analyzes how older Chinese immigrants interact with green spaces on the Gold Coast, Australia, and adopts the value of place as a conceptual framework to understand the relationship. Two qualitative methods, namely, in-depth interviews and travel diaries, were used to collect data. The results show that parks, as a place, play a crucial role in older Chinese immigrants’ ability to undertake outdoor activities. The relationship between green spaces and well-being can be classified into two themes. The first theme is concerned with how older Chinese immigrants perceive and experience green spaces. This finding indicates that green spaces can fulfill their values of keeping healthy, maintaining active lifestyles, and being social, all of which benefit well-being. The second theme relates to daily activities undertaken within green spaces. Issues of accessibility and personal preferences regarding activities complicate the relationship between green spaces and well-being. Good accessibility to green spaces is congruent with older Chinese immigrants’ values of being physically active, while difficulties in conducting preferred activities counteract these values which then generate negative perceptions of green spaces. Overall, there is great potential for understanding how personal values can inform the design of inclusive green spaces for minority or less mobile groups.

## Introduction

The number of urban inhabitants worldwide is expected to reach 6.6 billion by 2050, an increase of 52% from 2020, and of this 16% will be above 65 years old (United Nations, 2019). Human health and well-being are becoming priority goals for urban planners and city governors (United Nations, 2016), and urban green spaces are being regarded as an important urban environmental element that provides social, medical, and economic benefits (Bell et al., 2014; Douglas et al., 2017). For example, Lee and Maheswaran (2010) have indicated that urban green spaces promote well-being through reducing exposure to air pollution, noise, and heat. Neuvonen et al. (2007) and van den Berg et al. (2010) have shown how green spaces provide functions of psychological restoration and others, such as Hunter and Luck (2015), Sandifer et al. (2015), and Liu et al. (2019a), have demonstrated how green spaces facilitate physical activities and social engagement for residents. However, existing studies are often based on a premise that is centered around a general population. In other words, previous studies understand residents as a single and homogeneous group when investigating the relationships between green spaces and well-being (Hitchings, 2013). Obvious disadvantages of this approach include the lack of representativeness, as diverse minority populations (e.g., ethnic, age, or gender-based) are backgrounded and the influence cultural factors can have on the relationship between green spaces and well-being is ignored (Rishbeth et al., 2018). These gaps in research highlight the need for a further detailed investigation of the different benefits green spaces can offer to minority group members, as this may help urban planners and policymakers efficiently construct urban green spaces in the future (Douglas et al., 2017).

In this study, public green spaces describe natural areas in urban settings such as parks, gardens, woodlands, rivers, and beaches, which may incorporate natural, semi-natural, and artificial areas (Tzoulas et al., 2007; Bell et al., 2014). Public green spaces are an essential component of the urban green framework that can be accessed freely by the city population. These public green spaces are particularly important for minority and vulnerable residents who rely heavily on public spaces for leisure and recreation (Berney, 2010). However, in many cases, the public green spaces are distributed unevenly in urban areas, leading to urban residents not having equal opportunities to use public green spaces (You, 2016). Public green spaces have broader social significance in urban places (Barbosa et al., 2007) and, as a result, is a main concern within this research.

This research specifically investigates older people (in this research, older people are regarded as people who are aged 55 years old and above, based on Chinese statutory retirement age), as they are the fastest-growing age group worldwide (Wiles et al., 2012). Some institutions have published planning recommendations to help maintain the well-being of older people, including the milestone framework *Global Age-Friendly Cities: A Guide* published in 2007 by the WHO, which identified green spaces as a vital component of age-friendly cities (WHO, 2007). Interacting with green spaces on a daily basis can promote physical activity, decrease sedentary behavior, and reduce stress experienced by older people, positively impacting on their well-being (Wen et al., 2018). Another focus of this study is ethnicity, as the emergence of international immigration has resulted in an important demographic change globally that has seen the older population become more diverse racially and ethnically as a group (United Nations, 2019). As of 2016, in Australia specifically, over one third (37%) of older people were born overseas (Australian Bureau of Statistics, 2018a), with this number projected to increase rapidly until 2050 (FECCA, 2015). Among this minority-diverse group of older people, the older Chinese is one of the fastest growing groups, ranking as the third largest minority population nationally (Australian Bureau of Statistics, 2016a). Older Chinese immigrants who move to Australia later in life often aim to reunite with and support their adult children. They usually are not sure how long they will live in Australia. Immigration is the transnational movement with the intent of becoming a permanent resident of the destination country, while migration is the temporary movement. Their status of movement is in between and suspended, and thus they can be known as “parenting immigrants.”

Aging and living in a foreign country can lead to multiple vulnerabilities, including a decrease in mobility, weak mental health, high risk of social exclusion, and an increased need for recreational activities in green space (Byrne and Wolch, 2009). Language barriers and the inability to drive and/or take public transport can result in unfamiliar and stressful environments, which tend to increase the physical vulnerability of older Chinese immigrants (Tieu and Konnert, 2014; Lin et al., 2016; Luo and Menec, 2018). Considering the vulnerability of older immigrants, healthy aging of older immigrants is increasingly important. Healthy aging is defined as “the process of developing and maintaining the functional ability that enables well-being in older age” (WHO, 2015, p. 28). Green spaces that maintain older immigrants’ active lifestyles and independence could positively contribute to their healthy aging and overall well-being (de Keijzer et al., 2020). The multidimensional relationship that community members have with green spaces contributes to the unified understanding of well-being that integrates physical, social, and mental well-being into one concept (Ziegler and Schwanen, 2011). In this research, well-being is defined as a subjective phenomenon that describes people’s experiences of how well they are or how well they live (Fleuret and Atkinson, 2007; Ziegler and Schwanen, 2011).

Some scholarly work already exists on how minority groups use green spaces (Golledge, 1997; Byrne and Wolch, 2009). These studies demonstrate that people with different cultural backgrounds exhibit varying preferences for and engagement with different green spaces (Özgüner, 2011; Jay et al., 2012). For example, Byrne and Wolch (2009) showed that the White Americans may seek opportunities to exercise in green spaces, while Latinos sought to socialize. Green spaces express different meanings of place to people with diverse cultural and ethno-racial backgrounds (Egerer et al., 2019). Thus, it could be inferred that the benefit of green spaces may vary depending on the culture and the values of visitors. However, globally to date, little empirical research, including in Australia, has explored how cultural values influence the perceptions that minority group members have of green spaces and how their sense of well-being is gained. This study is focused on understanding green spaces and how they influence the well-being of older Chinese immigrants, within an Australian multicultural context through a lens situated in Chinese values and beliefs. The aim is to provide insights into why and how designing green spaces can better maintain the well-being of elderly immigrants in Australia.

Finally, the values of older Chinese immigrants are used to link the relationship between well-being and urban green spaces. Values are abstract concepts that are indicated by goals and accomplishments in daily activities (Tiberius, 2014). With this in mind, the specific values and the activities related to green spaces which are considered vital by older Chinese immigrants may have a role to play in their perceptions of green spaces and their well-being. These research stems from the hypothesis that people’s well-being and positive perceptions and attitude depend on what extent their values had been fulfilled (Sagiv and Schwartz, 2000; Schwartz, 2012). For older Chinese immigrants, the disjunction between current urban green places and their entrenched daily practices can impede their ability to fulfill their values, which can lead to negative perceptions of green spaces and thus also their well-being (Hodgetts et al., 2010). Therefore, investigating activities undertaken by older Chinese immigrants in green spaces assists in the understanding of the values, then perceptions, and well-being of members of this group.

The participants in the study are Chinese immigrants residing on the Gold Coast, Australia. Although Chinese immigrants usually settle in two major cities – Sydney and Melbourne, an increasing number have chosen to live in other cities since 2000 (Wang et al., 2018). On the Gold Coast, between 2006 and 2016, the number of Chinese immigrants rapidly increased from 2,945 to 8,408 (Gold Coast City Council, 2016). Chinese immigrants who were over 55 years old more than tripled and reached 1,694 in 2016, accounting for 20.1% of total Chinese immigrants on the Gold Coast (Australian Bureau of Statistics, 2016b). More specifically, Chinese immigrants have become Gold Coast’s top non-English speaking population in 2016, ranking up from a fifth position in 2006 (Wang et al., 2018). With the Gold Coast having an increasingly multicultural profile and rapid urbanization, it seems important to understand the immigrants’ interactions with the urban environment in order to meet diverse environmental needs.

As such, this research aims to examine important values held by older Chinese immigrants while exploring pathways that link public green spaces to their values and, therefore, well-being. The research questions are (1) to what extent do public green spaces influence the daily activities and well-being of older Chinese immigrants? and (2) how do older Chinese immigrants’ values influence their perceptions of public green spaces on the Gold Coast, Australia? Understanding these values may contribute to the development of future policies that work to design more inclusive urban green spaces.

## Methodology

**Figure 1:**
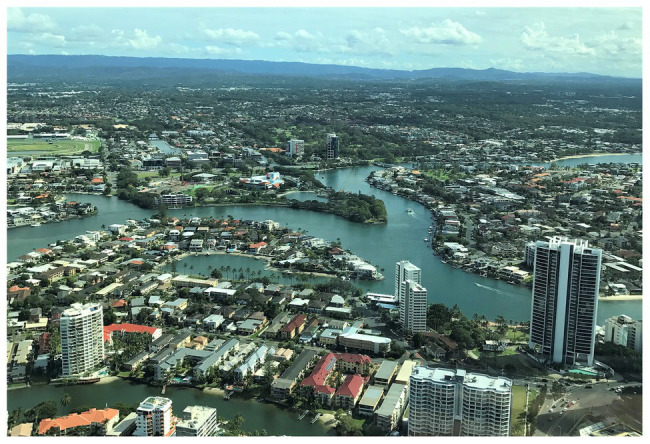
Aerial photo of Surfers Paradise on the Gold Coast facing west (photo by Siyao Gao).

Older Chinese immigrants living in the City of Gold Coast were involved in this study to explore how members of cultural and ethnic minority groups interact with urban green spaces. The Gold Coast is located on the eastern coast of Australia. Its 57-km coastline, spreading canals, and vast natural environments with a subtropical climate create a special urban form and park landscape (see Figure 1). The average temperature ranges from 16 to 29°C, with approximately 300 days of sunshine per year, and the mean annual rainfall depth is around 1,300 mm (Bureau of Meteorology, 2020). The parks on the Gold Coast accumulate to 19 million sq. m of land with various facilities for cycling, barbecuing, walking pets, and fishing (Gold Coast City Council, 2020). Residents on the Gold Coast have an abundance of green spaces, with 47.8 sq. m per capita. Among these public green spaces, the proportion of local parks, metropolitan parks, and regional parks are 57, 14, and 29%, respectively (Byrne et al., 2010). However, less than half of parks on the Gold Coast are accessible by public transport (Byrne et al., 2010). The mild climate, good air quality, copious amount of sunshine, and lifestyle continuously attracts an increasing number of immigrants to move to the Gold Coast, resulting in this city being one of the fastest-growing regions in Australia (Dedekorkut-Howes and Bosman, 2015; Bosman, 2016).

It is hypothesized that increased cultural and ethnic diversity will require a new strategy for urban green spaces. Therefore, this research examines the perspectives of older Chinese immigrants to determine features of urban green spaces that are relevant to their well-being. We draw from a mixed method combining travel diaries, mapping, and interviews to provide an in-depth exploration of green space visitation among older Chinese immigrants on the Gold Coast. First, travel diaries, as developed by Winters et al. (2015), were completed by participants to understand their activities, time-use patterns, and spatial travel behaviors within a single week. Then, with the help of an interviewer, the participants drew the travel routes to green spaces. Interviews were finally conducted, guided by the data of travel diaries and spatial patterns of activities. The interviews informed the quantitative data in greater depth. The quantitative data were used to understand the visitation patterns of older Chinese immigrants and also analyze and explore the participants’ experiences.

The participants completed travel diaries with the assistance of the interviewer. The interviewer sent reminder messages to the participants every 2 days to decrease the likelihood that the participants might miss any activities. The participants recorded details of each trip to green spaces, including the origins and destinations of the trip as well as other related temporal information, such as how they get to the destinations, their travel companion, and why they visit green spaces (e.g., exercise, recreational, or social purposes, etc.). The data from travel diaries were cleaned and entered into a SPSS database and used to describe the activity patterns of older Chinese immigrants. The participants were also asked to locate the green spaces they went to and the related travel tracks to these green spaces within their travel diaries. A total of 18 participants completed the maps (two couples participated, so the travel maps only show 16 houses). These mapping data were descriptively analyzed using ArcGIS software 10.3.

After the travel diaries and mapping exercises were completed, the first author conducted the interviews. Informed by the research objectives of this study, the research focused on the relationship between green spaces and well-being. Hitchings (2013) showed how qualitative approaches unveil subtle appreciations of the lifestyle and generate effective means of improving green space experiences; hence, this study adopted qualitative methods in the data collection and analytical process. The interviewees were prompted to provide details of their activities, movements, and experiences of visiting green spaces. Interview questions like “What did you do in green spaces before and after you immigrated to Australia?,” “What are the contributors and facilitators of conducting activities in green spaces?,” and “How do you perceive green spaces on the Gold Coast and in your hometown?” were raised. The well-being of older Chinese immigrants was self-rated in the process of the interviews. During the analysis of qualitative data, the proportion of participants with similar answers was calculated to show the overall situation.

Thirty participants completed the research at various places that they preferred, such as public parks, churches, and Chinese community centers. The interviews varied from 30 to 90 min. Mandarin, as the first language for both the interviewer and the interviewees, was the language used to conduct the interviews to minimize miscommunication in the research. During the analysis, the transcripts were translated into English by the interviewer and a proof-reader to ensure the accuracy of the translation and to avoid missing any cultural meanings as described by Suh et al. (2009). The participants were given pseudonyms to protect their identities while still denoting them as active and engaged subjects (Allen and Wiles, 2016).

This study used a purposive sampling strategy to collect the data (Liamputtong and Ezzy, 2005). In 2018, a preliminary study with six participants was conducted. By regularly attending Tai Chi classes and other social activities organized by Chinese organizations, the first author was able to establish a network of potential participants, with the assistance of local Chinese organizations. From February to May 2019, large-scale fieldwork was carried out. After gaining trust from potential participants, the first author recruited the participants by asking the participants’ willingness to join the research. Before commencing the interviews, all participants were given information statements and required to complete oral or written consent forms. In terms of the criteria used to recruit the participants, the Chinese retirement policy states that the statutory retirement age is 55 years old for female staff or 50 for female blue-collar workers and 60 years for males (Feng et al., 2020). Therefore, in this study, older Chinese immigrants who were 55 years old and above were deemed eligible to participate in the study. To ensure that the participants were able to provide reliable information about both their hometown and the Gold Coast, a minimum length of 6 months of stay on the Gold Coast was also a requirement of participant eligibility.

The analysis of the qualitative data was based on comparative methods (Corbin and Strauss, 2014) to develop the themes. The transcripts were read verbatim by the first author to mark each segment of meaningful data with a series of codes (Miles and Huberman, 1994). These codes were carefully scrutinized and constantly compared to identify and construct themes according to their similarities and discrepancies. Codes with similarities were grouped into a theme, which organized the raw data into conceptual groups. The initial coding list was created prior to collecting qualitative data based on the research question and literature, including key language such as “green spaces,” “physical activities,” “social activities,” “relationship between green spaces, activities” and “well-being,” and “relationship between green spaces and well-being.” In the process of coding, according to the concept of values that were embodied in the goals of visiting green spaces, “keeping healthy,” “active lifestyle,” and “community inclusion” were included as emergent codes. Finally, these codes formed core themes that offered some insight into the relationship between green spaces and well-being. The transcripts were analyzed with the aid of the qualitative software NVivo 12.

## Results

Overall, the sample included 11 males and 19 females. Their ages ranged from 61 to 83 years old (the average age was 69.4 years old), with 70% aging between 65 and 80; at the time of the interviews, their length of stay in Australia ranged from 1 to 15 years (the average length was 5.5 years). Among these participants, 17 participants lived with both their spouse and children’s families, 10 participants lived with their spouses only, and three lived with their adult children only, without a spouse. These participants lived in various types of neighborhood with different levels of accessibility to green spaces. The neighborhood types where the participants resided were characterized as low, medium, and high advantage using the Socio-Economic Indexes for Areas. This index was determined by income, education, employment, occupation, and housing characteristics of the neighborhood (Australian Bureau of Statistics, 2018b). In this research, out of the 18 participants who provided their residential addresses, seven of them lived in high-advantage neighborhood environments, and the remaining 11 participants lived in medium-advantage neighborhood environments. Regarding the participants’ neighborhood environment, according to the standards proposed by the Gold Coast City Council (2019), six participants lived in high-density zones with high accessibility to public transport, community facilities, and green spaces, while the remaining 12 participants lived in suburban areas far away from public infrastructures.

Three themes were established from the research. The first theme centered around older Chinese immigrants’ daily patterns of visiting green spaces. The second focused on contradictions between preferences of activities and barriers associated with green space visitation. Finally, values embodied in visiting green spaces, including values of keeping healthy, maintaining an active lifestyle, and being social, are analyzed.

### Patterns of Everyday Contact With Public Green Spaces

Descriptive statistics were used to depict the general pattern of green space visits undertaken by the participants. The travel diary was completed by 30 participants who documented 380 trips within 7 days. The result indicates that public green spaces are the main destinations for older Chinese immigrants. Among these trips, nearly one-third of trip destinations (101) were to parks near their homes, and all of these trips were made on foot. A total of 45 trips were to other parks, which accounted for 11.8% of all trips. The participants went to other parks by various means of transportation, including private cars, walking, and public transport.

**Figure 2:**
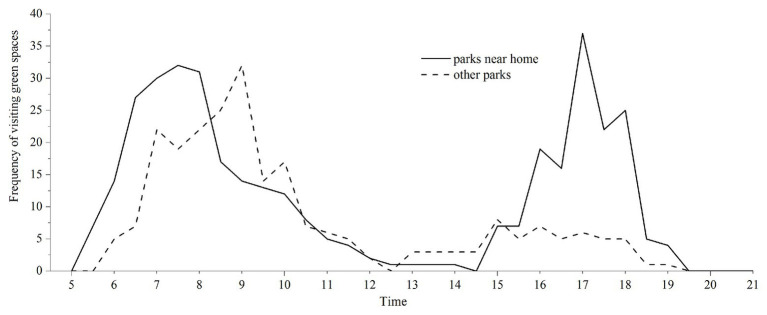
Timing of visits to urban green spaces.

The patterns highlighted, based on the time of visits to the green spaces within a day, indicated two clear waves of visiting hours (Figure 2). Mornings (6:00–9:00) were the most preferred time, according to these participants, to visit both parks near home and other parks, while late afternoons (16:30–18:00) were popular for visiting parks near homes only. Visiting green spaces in the morning may be attributed to activities such as Tai Chi or social activities often being considered and therefore organized as morning activities. Additionally, 66.7% of participants expressed their willingness to visit the spaces in the evening.

As for the type of companions that made these visits with the participants, the participants mostly favored visiting public green spaces with their spouses (47.6%), alone (21.9%), or with friends (19.8%). Visiting with pets or family was less favorable, with these companions only preferred by 7.5 and 3.2%, respectively. The result indicates that green spaces offered a place for them to conduct activities independently. As Bei (female, 64 years old, living in Australia for 3 years) pointed out:

“I can’t drive or take buses here. The small park near my house is a place for me to visit independently. I can go to that small park at any time without asking my son to drive me there. I can enjoy time by myself.”

**Figure 3:**
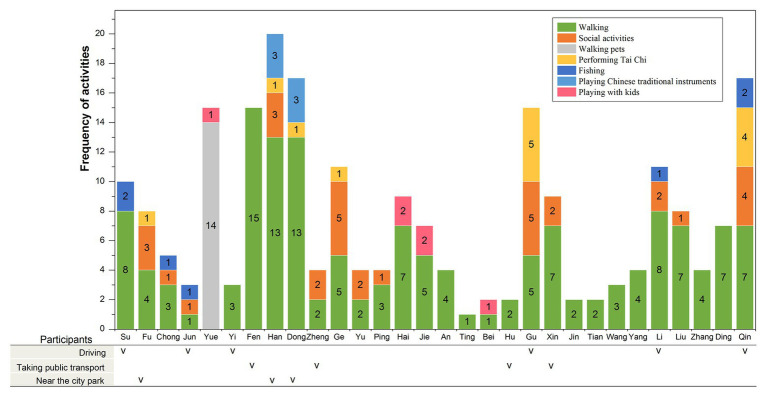
Individual activity patterns of participants (“v” indicates that the participants have the ability to drive or take public transport on the Gold Coast or live near the city park – these groups of people are regarded as having high mobility; participants without a “v” have limited mobility).

The individual activity patterns of the 30 studied participants in urban green spaces are shown in Figure 3. Walking was by far the most popular activity undertaken in public green spaces (66.5%), while social activities were the second most frequent (14.2%). Social activities included having conversations, exercising, and having recreational activities together. The proportions of those who walked pets and played Tai Chi were 6.4 and 6.0%, respectively.

### The Influence of Preferred Activities and Accessibility

The high accessibility of green spaces encouraged the participants to visit these spaces and conduct more physical activities. Yi (male, 71 years old, living in Australia for 1 year) noted:

“The park is near my home, only 2 minutes’ walk. It’s really convenient. If I have time, I will go to that park and sit for a while.”

**Figure 4:**
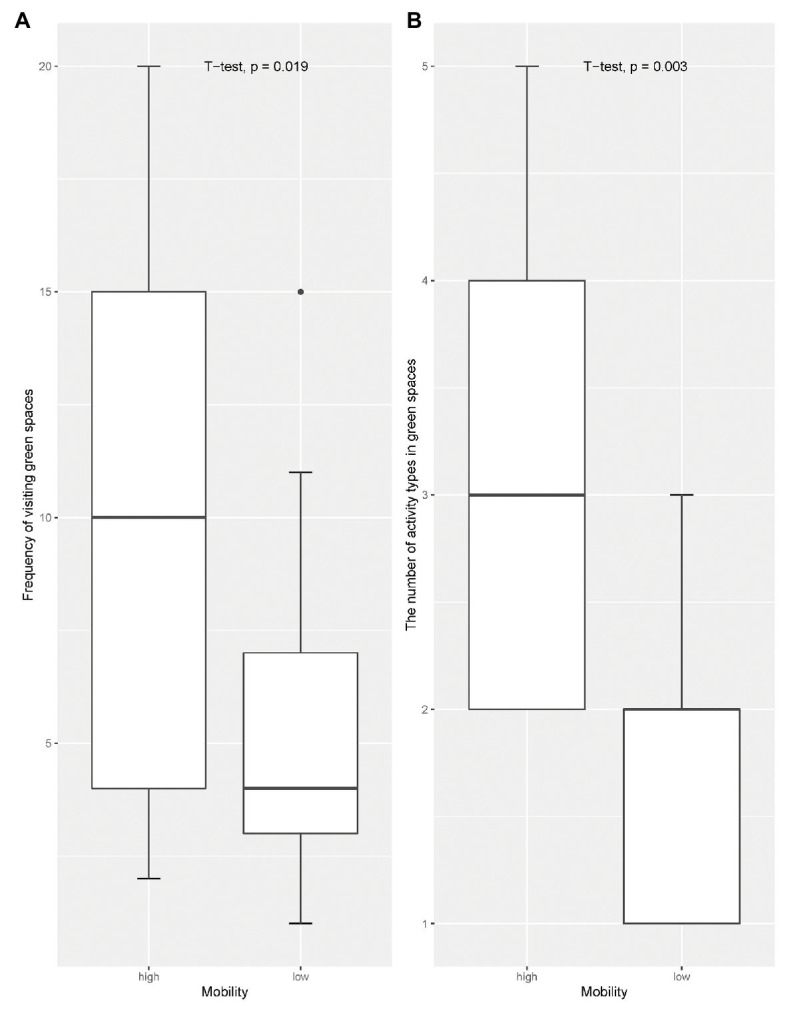
Box plot illustrating the frequency of visits to green spaces and the types of activities undertaken with reference to the participants mobility level. **(A)** Comparing frequency of visiting green spaces between high and low mobile groups. **(B)** Comparing the number of activities undertaken in public green spaces by high- and low-mobility groups.

The participants who were able to drive or take public transportation on the Gold Coast or lived near city parks that hold Chinese activities are classified as having high mobility. Others are seen as having low mobility. Differences between low and high mobility were examined using *t*-tests. The comparative analysis between the high- and low-mobility groups resulted in significant differences (Figure 4). Compared to participants who were considered “less mobile,” those who were “more mobile” (*n* = 13) had a higher frequency of visiting green spaces (*p* = 0.019) and conducted more types of activities in green spaces (*p* = 0.003). Yue was an exceptional case. Yue had low mobility but had to walk the dog twice a day, which significantly increased her frequency of visiting green spaces.

Issues of accessibility and personal preferences regarding activities complicated the relationship between green spaces and well-being. For instance, older Chinese immigrants preferred green spaces that supported their ability to partake in Chinese activities. On the Gold Coast, all (100%) participants reported that they still retained a willingness to continue their previous lifestyle and physical activities in green spaces, such as performing Tai Chi, square dancing, and singing. Gu (male, 63 years old, living in Australia for 2 years) stated his experience:

“I like performing Tai Chi. I practiced Tai Chi for nearly 10 years before I moved to Australia. Now, I’ve joined a Tai Chi club, and I can continue to practice it.”

**Figure 5:**
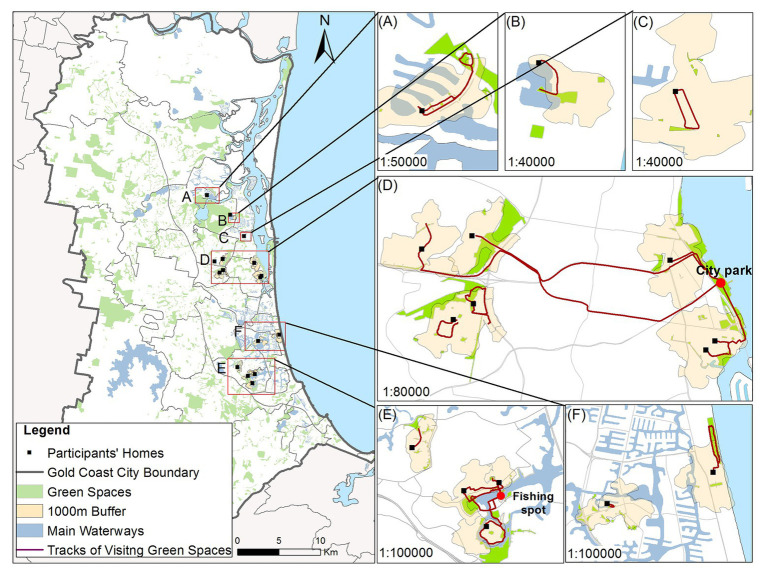
The participants’ location and their travel maps to green spaces on the Gold Coast. (**A**-**F**) show detailed participants’ location and travel maps. (source: image produced by the author with spatial information obtained from the Australian Bureau of Statistics and the Gold Coast City Council).

As indicated in Figure 3, one-fifth of the participants performed Tai Chi within 7 days before the interview. Two participants, Dong and Han, a couple, played traditional Chinese instruments in the park near their homes. The participants revealed that the activities that they preferred, such as Tai Chi and square dancing, were mostly clustered in the city parks on the Gold Coast. The participants’ residences were scattered across the Gold Coast, which meant that few of the participants were able to visit their preferred parks. Figure 5 shows the travel maps of the 18 participants within a 1,000-m zone highlighted on each map. It showed that the participants’ behavior in visiting parks and their travel tracks depend on the distribution of parks within a 1,000-m zone (see Figures 5A–C,E,F). They could walk to the parks near their home, which indicated that the accessibility of parks could partly meet the participants’ needs. However, they had lower accessibility to their preferred park. As shown in Figure 5D, people who were able to drive or take public transportation could go beyond the 1,000-m zones, visit the city park, and participate in dancing, Tai Chi, and other preferred activities, while others were limited in their ability to do this, as they could only visit neighborhood green spaces within their 1,000-m zone. More than one-half (57%) of the study participants reported that they could barely conduct the physical activities they preferred. For example, as Yue (female, 61 years old, living in Australia for 2 years) complained:

“Actually, there is a park that has activities such as square dancing and Tai Chi. Many older Chinese people do these activities there. But I can’t go there by myself. So, I can’t join them.”

Older Chinese immigrants had accessibility to visit green spaces near their homes. The fact that they were unable to visit the parks that they preferred negatively influenced their perceptions on green spaces on the Gold Coast.

### Values Embodied in Green Space Visitation

Three main types of values pertaining green spaces emerged through the analysis of the interviews, namely, keeping healthy, maintaining active lifestyles, and being social.

#### The Value of Keeping Healthy

The participants expressed at length that their most essential purpose of visiting public green spaces was to remain healthy, which embodied the participants’ values of keeping healthy. The participants perceived that green spaces provided a healthy landscape and place to undertake exercise. First, all (100%) participants noted that exposure to green spaces provided pleasure and positive feelings. They pointed out the positive elements that formed a healing landscape for them, including what they saw, heard, and felt, such as the quiet and peaceful scenery, lawn, tree-shade, birdsongs, temperature, and winds. They showed sensitivity to the landscape and awareness of its health benefits. The participants developed a sense of improved health when they visited green spaces, congruent with their values of maintaining health, and thus this also generated positive perceptions of green spaces. As Hu (female, 70 years old, living in Australia for 9 years) pointed out:

“I am happy to live here. The parks here make me feel close to nature. They provide an open space where I can sit and gaze into this landscape. I can feel the warm sunshine, see the beautiful scenery, and hear the birdsong, which helps me escape from annoyances. It is definitely good for my health!”

The participants also perceived green spaces as providing opportunities to conduct physical activities based on their values of keeping healthy. According to the data drawn from the travel diaries, nearly all (93%) the physical activities or exercises undertaken by this group were conducted in urban green spaces, while the remaining 7% of physical activities were conducted in community centers or churches. This result alludes to the essential role of green spaces, as these participants heavily rely on these spaces for their physical activities. Fu reported:

“I only do some simple exercises in the park near my home, such as walking or doing some stretching. Green spaces enable me to walk more. It’s easy for me to walk 1 to 2 kilometers in the parks. It’s good for our health. If I stay at home, I think I won’t be able to go out anymore. That means my health may collapse. So, I go to the park every day.”

However, older Chinese immigrants pointed out that green spaces on the Gold Coast cannot completely meet their needs of keeping healthy as they cannot always undertake particular Chinese activities that act as a means to fulfill their values of keeping healthy, such as performing Tai Chi, square dancing, and singing. It was discovered that few parks on the Gold Coast could meet such needs. The participants (57%) who could not undertake their preferred activities made modifications to compensate for these barriers in order to continue their ability to participate in physical activities. To some extent, walking was seen as a complement to their physical activity need. Because of the differences between the green spaces in China and in Australia, the participants fulfill their values of being active and healthy on the Gold Coast to a lesser degree, which has generated negative perceptions of green spaces as older Chinese immigrants are unable to continue the lifestyle that they had in China. Ping (female, 65 years old, living in Australia for 7 years) made the following comparison:

“In my hometown, there are always various activities, such as square dancing and choir singing. I can participate in these activities. I always want to find similar activities here, but it’s difficult. I only walk here, nothing else.”

#### The Value of Active Aging

Another reason for which the participants visited a green space was to fulfill their values of maintaining an active lifestyle. Due to their reduced social network and limited mobility, 77% of the participants pointed out that they had fewer formal or informal activities after migration. However, all (100%) the participants visited green spaces at least once a week. The fact that all the participants visited green spaces reinforced their desired to have an active lifestyle in later life. They prioritized going to green spaces as a regular daily activity to spend leisure time and escape boredom. They perceived experiences with green spaces to be integral in experiencing a fulfilling day. As Fu noted:

“I go to the small park to walk and do some exercise twice a day. Once is in the morning and the other is after my dinner. In China, I have lots of physical or social activities, but in Australia I have no place to go. So, going to the nearby park has become a thing for me; otherwise, I have nothing to do and I am always at home.”

During the interviews, 70% of the participants compared the activities that they could undertake between their hometowns and the Gold Coast, indicating that they preferred the lively atmosphere in green spaces, mostly in China. Parks there were seen as public places which held various activities and created a vibrant place, as expressed by Ding (female, 65 years old, living in Australia for 5 years):

“The parks in my hometown are lively; there are many people walking and chatting with each other. There are also many activities, such as singing, dancing, and Tai Chi. It’s a little bit crowded but lively. When I saw other older people doing these activities, I felt that I also had an active lifestyle.”

More than half of the participants (60%) were unsatisfied with the green spaces on the Gold Coast during their green space visitation. They perceived that the monotonous landscape of green space could not fully meet their values of being involved in an active environment as Tian (female, 68 years old, living in Australia for 4 years) noted:

“Although the environment is good, it is boring. There is no other scenery I can see. The trees, flowers, and large lawn are always the same. You can see a few people walking or running. I feel bored.”

#### The Value of Being Social

The third important function of green spaces as described by the participants is how they enable the construction of social connections with people and communities. A majority of participants (67%) agreed that going to public green spaces was a useful way to develop social connections with friends and neighbors. The desire to build social networks reflected the participants’ values of being social. The participants were more likely to cluster in green spaces which were visited by other Chinese people. Two popular green spaces are shown in Figures 5D,E. Figure 5D and the interview responses indicated that older Chinese immigrants met their friends in the city parks on the Gold Coast, initially spontaneously and, thereafter, intentionally and regularly. They organized Chinese social groups in public green spaces and managed to participate in these social activities. The participants indicated how this increased positive feelings of well-being. With the increased number of Chinese immigrants interacting together in these spaces, the activities have increasingly gone beyond social function as the development of other group activities, such as Tai Chi, choir singing, and formed bands have also emerged. Figure 5E depicted a fishing spot favored by older Chinese immigrants. As stated by the participants, fishing, as an activity, encouraged them to meet and chat with each other as they exchanged fish and fish dish recipes and thus also enabled the participants to build social networks.

However, the participants perceived Gold Coast green spaces as places where certain barriers prevent their ability to conduct their preferred social activities. All (100%) the participants expressed the difficulties of interacting with their neighbors because of the language issue. They seldom start a conversation with their neighbors and often do not establish stable and deep relationships, as expressed by Xin (male, 76 years old, living in Australia for 9 years):

“I sometimes see some neighbors in the park. But we don’t communicate much. It’s annoying because I have many words to say but I can’t express myself. I have learnt some simple sentences to communicate with them, but that’s not enough. It’s a pity that we don’t have any in-depth communication.”

Although they struggled to develop strong social networks, the interview confirmed that constantly visiting public green spaces helped these participants generate a sense of belonging to their place of residence. Within these green spaces, older Chinese immigrants have the opportunity to contact neighbors regularly, such as by greeting, smiling, or recognizing faces. Close to three-quarters of the participants (77%) expressed that, through this regular contact with their neighbors, they gradually developed an attachment to the neighborhoods, which positively influenced their well-being, as denoted by Dong (male, 67 years old, living in Australia for 4 years):

“I go to the nearby park every day, and I can see some familiar faces. They look very friendly and warm-hearted. They show me how to use fitness equipment; our dogs can play together. I feel very happy that I can live in that neighborhood. I feel I am a member of that place now.”

Overall, values of older Chinese immigrants have been extracted from the interviews. Their perceptions of green spaces are informed by their values and therefore can have effects on their well-being.

## Discussion

With the aging population in urban areas becoming more diverse, a key issue in research, for urban planners, is how services and activities can be provided to residents with various needs in public green spaces. This study explores the diverse and complex interrelationship between green spaces, perception, well-being, and values among older Chinese immigrants in Australia. This paper utilizes a mixed method as it combines the use of travel diaries, mapping exercises, and interviews to draw data. This method identified patterns in the participants’ interactions with green spaces and revealed their preferences in green spaces. As their preferred green spaces are clustered on the Gold Coast, poor accessibility was a barrier for older Chinese immigrants trying to engage with public green spaces. Their interaction with green spaces embodied their values of keeping healthy, having an active lifestyle, and fostering social connections. The concept and the design of urban green spaces on the Gold Coast impede older Chinese immigrants’ ability to fully express their values, which then negatively influences their perceptions on green spaces.

First, the results from the travel diaries highlight the spatio-temporal patterns apparent in older Chinese immigrants’ behavior and interaction with green spaces and physical activity. Older Chinese immigrants’ willingness to visit green spaces at dusk may be attributed to a traditional belief in China, which suggests that taking a stroll after dinner is good for health (Cerin et al., 2013). These findings indicate the importance of managing green spaces to accommodate for use of the space during the night. The poor lighting in residential areas creates insecure environments that impeded upon the participants’ ability to walk in green spaces at night. Therefore, developing high-quality paved trails that are clearly lit would likely encourage older Chinese immigrants to maintain their active lifestyle, as this would enable them to feel secure and visit green spaces in the evening. For older Chinese immigrants who cannot walk independently and/or are unable to take public transport or drive, green spaces near their homes play a significant role in their ability to achieve their physical activity goals. This finding is also congruent with a previous study which found that urban green spaces can be especially significant for vulnerable groups (Maas et al., 2006). For people with mobility issues such as other immigrants, disabled residents, or people who suffer from mental health issues, public green parks, especially within their neighborhood, are key places that help to promote social networks and community engagement. Staff related to green spaces management therefore can play a positive role by helping minority groups access the major city parks where various activities are held. Improving the quality of walking tracks and illuminating the road could also help promote a sense of security when visiting green spaces. Public green spaces are important features for these vulnerable groups as these enable them to engage with their society. Therefore, when creating neighborhood governance policies, urban green spaces should be taken into consideration.

Second, older Chinese immigrants are more likely to visit urban green spaces either with their spouse or alone, but seldom with family. Older Chinese immigrants usually depend on the assistance of their adult children for transportation, shopping, and medical care (Guo et al., 2016). As a result, visiting green spaces can be seen as a means to fulfill their desire to conduct activities independently. Older Chinese immigrants often visit green spaces independently and enjoy their own leisure time, which requires green spaces to have high security. Limited mobility and safety concerns, such as worries about getting lost, can negatively influence their interaction with green spaces. Based on this finding, urban planners and green space managers should support residents who are frail or who have mobility limitations to enable them to visit green spaces more freely. Several strategies can be applied. One of these is to organize activities, with the help of public service organizations or immigrants’ associations, to enhance the accessibility to the preferred activities of members of this group in these green spaces, and to help encourage older Chinese immigrants to feel positive about visiting green spaces. Another is to improve the sense of security in green spaces to encourage vulnerable residents to visit. This could be done through methods such as designing disability access and monitored green spaces with formal or informal surveillance. For those people who have mental health issues or language barriers, a sufficient sense of security encourages them to visit green spaces and ultimately benefit their health and well-being.

Green spaces were also important places for older Chinese immigrants as they helped to develop social networks, which then provided opportunities for them to perform traditional collective Chinese activities. However, difficulties in accessing these particular green spaces hindered their ability to participate in activities. In China, high-density urban development makes it possible for residents to walk or take public transportations to destinations. Older Chinese immigrants are used to walking to nearby green spaces to participate in activities. However, the urban traffic pattern in Australia heavily relies on private vehicles. On the Gold Coast, 88% of daily trips are made by cars, while only 7% of trips are by walking and the remaining 3% are by public transport (City of Gold Coast, 2013). Major thoroughfares and an abundance of canals serve as neighborhood boundaries. Large expanses of tract housing in suburban areas restrict the residents’ ability to visit other parks in the city center or outside of their neighborhoods. Participants with lower mobility are limited to visit green spaces that are far from their home, only able to visit local-neighborhood green spaces. It can be deduced that the accessibility to infrastructures affects the travel routes and visitation to green spaces, which is closely related to the neighborhood type in which they live. With this in mind, further studies of how accessibility to green spaces in urban areas can be increased should be considered. With the number of immigrants increasing, collaborating with Chinese organizations to better understand the needs of these minority group members so that culturally sensitive planning can take place should be taken into account in future development and renovation plans for urban green spaces.

In 2014, the average neighborhood green space area in China was 12 m^2^, compared to 154 m^2^ in South East Queensland, Australia (Wang et al., 2015). Urban green spaces are also socially mediated ecologies, developing within a particular culture and social ideology. In Australia, green spaces differ from those in China by size, the ornamental plants, the design, and the facilities (Wang et al., 2015). These social, cultural, and ecological differences shape how older Chinese immigrants perceive and utilize green spaces (Byrne and Wolch, 2009). The results also find that participants are aware of the differences in the soundscape between the Gold Coast and their hometowns. The birdsongs mentioned in the interviews and the participants’ described tranquil experiences in green spaces helped to form a soundscape for public green spaces (Liu et al., 2019b; Shu and Ma, 2020). Therefore, further studies are needed to investigate how the older immigrants perceive the soundscape and how different cultural factors affect their relationship with the soundscape in green spaces and their well-being. The green spaces present within this research are informed by western culture, which assumes that visitors focus on individualism and the quiet enjoyment of nature (Byrne and Wolch, 2009). Older Chinese immigrants, however, prefer lively environments of green spaces. The particular lifestyle and activities of older Chinese immigrants influence the way they express their values in relation to green spaces. Although they praise the landscape in green spaces and its physical benefits to their well-being, the participants had negative perceptions of green spaces because of their own cultural values, which resulted in diminished social and mental well-being. The benefit of approaching greenness is reduced when the green space cannot meet the values of residents. Therefore, in order to maximize the positive functions of green spaces for older Chinese immigrants, values held by this group should be firstly identified.

With regards to urban planning, although many previous studies call for inclusive urban planning for multicultural cities (Rishbeth, 2001), few strategies have been implemented into different measures and programs. With the increase of ethnic minority groups in urban areas, urban planners must also be sensitive to the significance of minority ethnic groups as a part of local society. For example, the voices of immigrants could be taken into consideration in the process of designing green spaces and city governance.

The limitations of this study also warrant a mention. First, the research recorded 30 interviewees’ travel behaviors; therefore, their patterns of green space visitation may not be representative of a full picture that reflects all older Chinese immigrants’ living conditions on the Gold Coast. However, the analysis of the interviews still offers great insight into older Chinese immigrants’ perceptions of urban green spaces and the role that cultural values play in the relationship between green spaces and well-being. Regarding the participant recruitment process, all the participants were recruited through Chinese community centers; therefore, most of the participants were relatively healthy, active, and capable of participating in outdoor activities. The most vulnerable individuals who were less mobile were therefore underestimated. Second, this research treats the older Chinese immigrants as a homogeneous ethnic group and ignores the variety of their demographic characteristics, background, and living conditions in their hometowns. More explicit breakdowns could enable these factors to provide further insight into their interactions with green spaces after immigration. Third, this study only discusses the values of older Chinese immigrants in order to construct an inclusive urban green space; however, future studies could compare the experiences of older Australians and other older minorities. Finally, this research only focuses on parks and ignores other types of greenness in urban areas such as the green spaces along the road or surrounding homes and the private green spaces within the participants’ houses.

## Conclusion

This research examines identifiable patterns pertaining to green space visitation, with specific focus on older Chinese immigrants and the relationship between green spaces and their well-being on the Gold Coast. The results indicate that green spaces maintained the well-being of older Chinese immigrants. For older Chinese immigrants, Chinese-related activities significantly influence their perceptions of green spaces. However, the preferences for activities and a lack of accessibility to green spaces also negatively influenced older Chinese immigrants’ ability to perform their values of keeping healthy, being social, and being engaged. The fact that the values of older Chinese immigrants are less fulfilled in green spaces on the Gold Coast indicates a contradiction among green space provisions, demand, and utilization. The findings highlight the importance of activity management and multicultural planning and design of urban green spaces.

This study adopted a mixed method combining the travel diary, mapping travel routes, and in-depth interviews to understand the participants’ behavior and experiences. The mixed method helped compensate for independent methodological weaknesses. The travel diary and the mapping exercise aided in the visualization of the participants’ travel routes and provided a window of insight into everyday situations and interactions that occur within green spaces. The combination of interviews strengthened the understanding of the ways in which participants experience green spaces and the meaning and functions that drive their motivation to conduct certain activities in green spaces. The participants revealed dimensions of their social context that influenced their sense of well-being when they visited green spaces, such as the language barriers and experiences of stress that stem from insecurity.

This study highlights how values play a crucial role in how the relationship between green spaces and well-being is understood. The theory of value provides urban planners and policymakers with a better understanding of which elements of the urban environment are important to residents and therefore helps to shape priorities for policy and maximize the benefits of environments. Cultural variation should be taken into account during the decision-making process, which requires participatory or interactive planning and management between residents and urban planner. Green space planning and management should contribute to green spaces with serious consideration of the physical and social needs and expectations of vulnerable groups and residents with diversified values.

This research sheds light on the accessibility of urban green spaces and the provisions that enable age-friendly community planning. Increasing accessibilities of various levels of green spaces, especially for those who organize cultural activities, could significantly increase the benefits of those green spaces. It also needs to be mentioned that the implications of this research are not just for older Chinese immigrants but also for other vulnerable groups who are not being effectively served by urban parks. Immigration and the aging population have altered the demographic compositions in urban areas, which had resulted in complex demands being sought in urban green spaces. When considering the increasing number of older people and the ethnic minority groups, their preferences in recreational activities and their social needs should be considered in future urban green space planning. More attention should be given to cultural elements and facilities that promote exercise and social networks within the provisions and design of green spaces. Intensifying culturally sensitive strategies in urban planning and design is important. For a long-term policy, being sensitive to the values of residents is necessary for urban planners if they wish to develop more appropriate facilities that meet the needs of those living within a dynamic demographic structure.

## Data Availability Statement

The raw data supporting the conclusions of this article will be made available by the authors, without undue reservation.

## Ethics Statement

The studies involving human participants were reviewed and approved by Griffith University Human Research Ethics Committee. The ethics committee waived the requirement of written informed consent for participation.

## Author Contributions

SG contributed by designing the manuscript, map designing, and structuring and writing the manuscript. CB and KD contributed by reviewing and editing the manuscript and supervision and project administration. All authors contributed to the article and approved the submitted version.

### Conflict of Interest

The authors declare that the research was conducted in the absence of any commercial or financial relationships that could be construed as a potential conflict of interest.
